# Lost in digitalization: on the vanishing of the individual patient in the medicine of the future

**DOI:** 10.1186/s13010-026-00242-1

**Published:** 2026-11-10

**Authors:** Marco Stier

**Affiliations:** University of Münster, Münster, Germany

**Keywords:** Bioethics, Loss of the individual, Digitalization, Bias, Telemedicine

## Abstract

Although digital medicine promises unprecedented individualization through data-driven precision, this paper argues that the opposite trajectory could emerge. Unless medicine proceeds with caution, the patient risks being reduced to a statistical abstraction, removed from the physician’s moral and perceptual grasp, as digitalization accelerates. In the article, I discuss three possible distortions that could contribute to this trend: First, patients could be reduced to quantifiable data points through datafication. Second, several forms of bias may contribute to the homogenization of patients or the neglect of individuality. Third, virtualization in telemedicine may foster the fragmentation and depersonalization of the patient–physician encounter. Together, these developments risk diminishing rather than enhancing personalized care. The paper therefore calls for critical vigilance to ensure that technological innovation remains anchored in attentiveness to the unique, embodied individual—such that, in pursuing precision, medicine does not lose sight of the very person it seeks to serve.

## Introduction

Historically as well as systematically, the clinical practice of medicine has been understood as directed toward the particular sick person, rather than merely toward disease categories or abstract patient populations. To put it in the words of Francis Peabody: “The treatment of a disease may be entirely impersonal; the care of a patient must be completely personal.” [1] This understanding of the main goal of medicine is also at the heart of patient-centered care [2]. Yet recognizing and responding to individual needs can be challenging, given the inherent limitations of human knowledge, the often nascent state of technological development, and, not least, the fragmentation caused by healthcare systems. Now, in the era of digital medicine, many assume that physicians will finally be able to offer truly tailor-made treatments. While this hope is certainly not unfounded, an alternative perspective suggests that the opposite may also occur. If medicine proceeds without caution, it may not only fail to move closer to the patient but may ultimately lose sight of the individual altogether. In what follows, I will explore how digital technologies, despite their promise, may not reduce—but may even increase—the distance between medicine and the patient.

A note may be in order at this point. The deindividualizing tendencies of digital technologies in medicine that I am going to discuss are in no way necessary or inevitable, nor are my claims based on empirical evidence. My point is rather a warning than a prediction. If what I present as *one possible* future of medicine does not come to pass, so much the better. Yet it would be reckless to assume that what I am going to point to in this paper poses no real threat.

One common objection to my thesis—that medicine risks losing contact with the individual patient in the process of digitalization—is as follows: No one seriously intends to replace physicians entirely with artificial intelligence (AI) systems, so the caring physician will always have the final say. This may be true in principle. Yet there are good reasons to question this assumption. Four, in particular, deserve attention. First, technological developments rarely unfold exactly as expected. Second, AI systems are already too complex and intricate to be “corrected” by a simple human mind. Third, commercial and institutional interests are likely to shape clinical practice. And fourth, the well-known “automation bias”—the tendency to assume that “the computer knows best”—will often result in AI effectively having the last, and perhaps only, word.

In developing my argument, I will proceed as follows. I begin with a brief historical sketch of the “personalized medicine” approach, which originally started with the concept of “personalization”, later shifted its focus to “precision”, only to come back to personalization in the digital era. Against this background, I will introduce three possible “distortions” that threaten the ideal of a truly individualized medicine: datafication, bias, and ​the distancing and virtualization of the patient–physician relationship.

Today, datafication almost always means digital datafication, and it tends to encompass nearly every aspect of life—even phenomena that no one would have thought to “quantify” just a few years ago. Yet it must be remembered that data, contrary to popular belief, are not objective reflections of reality. They do not show the world “as it is”. Even if data were somehow objective, they would still not constitute knowledge or medical wisdom. Moreover, the algorithms that process data and render them operational are often opaque.

The second distortion arises from bias. The problem of bias is far from new; however, beyond producing incorrect or discriminatory outcomes, bias also entails the loss of aspects that define the individual patient. In clinical practice, a biased result is not merely wrong—it diminishes the patient’s uniqueness. This can manifest in two specific new forms of bias, which I will describe below.

The third distortion comprises three specific “losses” arising from the virtualizing distance between patients and physicians in telemedicine. As the “spatiotemporally integrated treatment situation” (as I call it) becomes split and fragmented, the *body* as lived body, the treatment *focus*, and important *context* are at risk of being lost.

In offering these reflections, I do not wish to diminish the value or potential of digital technologies. Rather, I argue that medicine must remain vigilant not to lose what it has always held dear: attention to the individual.

By now, “digital medicine” likely needs little further explanation. Concepts such as big data, AI, clinical decision support systems (CDSS), digital phenotyping, AI image analysis, and deep learning have been widely discussed for several years. For the purposes of this essay, I will simply define digital medicine as the use of digital technologies—including sensors, mobile applications, data analytics, and AI—to measure health, support clinical decision-making, and deliver personalized care.

Before proceeding, one further question must be answered: what is meant by “individual” in what follows? This word can refer to a lot of different things—and in fact, it does so throughout this essay, even though what is at stake comes down to a clear, circumscribable problem. To get a better understanding of my usage of the concept, four levels can be distinguished. First, the usage as a noun: this individual (a person). Second, the usage as an abstractum: the individual (in contrast to a group). Third, “individual” as a subsuming, nominalized adjective: the individual traits, physical and mental properties, etc. that make someone a distinctive and unique person. Fourth, “individual” as an adjective in connection with a noun: an individual need, hope, or even body. Granted, all this may seem to constitute a conglomerate of aspects too diverse to be useful in a philosophical analysis. In light of the main problem being discussed here, however, they are just variations and explanations of a single theme: the individual patient.

## The promise of personalization through digitalization

Patricia Baird’s passionate appeal that “[w]e need to see our own genetic individuality as a potential origin of disease”, and her call to incorporate this insight into disease prevention [3], still resonates in today’s hopes for digital medicine. Nearly a decade after Baird, the term “personalized medicine” was coined by Langreth and Waldholz [4], although the idea of adapting medical treatments to the specific characteristics of individual patients can be traced back at least to the 1960s [5]. It is therefore unsurprising that current initiatives are often referred to as “digital personalized medicine” (DPM) [6–8]. Interestingly, advocates of digital medicine are now seeking to fulfil a promise that the original endeavour of “personalized medicine” (PM) never actually made: the *individualization* of specific medical treatments. Many have argued that “personalized medicine” was a misnomer from the start, since it was never intended to focus directly on the individual patient [9]. As the President’s Council of Advisors on Science and Technology [10] noted, personalization did “not literally mean the creation of drugs or medical devices that are unique to a patient.” Rather, the vision was one of greater *precision*—a medicine capable of improving diagnostic and therapeutic accuracy. In a second step, such precision was expected to yield better outcomes for individuals. Yet even this was questioned, because a medicine that builds on genetic data but fails to include clinical, social, and environmental features, could easily lead into a “depersonalized” medicine [11]. Despite the conceptual shift from personalization to precision, the aspiration for truly individualized treatment never entirely disappeared. The hope persisted that once medicine possessed “comprehensive information about each patient’s unique genetic makeup,” physicians would be able to select “the best specific diagnoses and therapeutic methods for that individual” [12]. Medicine, it was said, should transcend the “one-size-fits-all” model and aim for the “prescription of specific treatments and therapeutics best suited for an individual, taking into consideration both genetic and environmental factors,” thereby ensuring that “the right treatments are given to the right patients at the right time” [7].

From this still short history of personalized medicine, three lessons emerge: ​ (1) “Personalization” originally denoted “precision”, but the desire for *genuinely* individualized medicine was present from the beginning. (2)​ ​Although PM originated in genetics, it has become clear that genetic information alone is insufficient; a truly individualized medicine requires far more comprehensive data about each patient. (3) ​The precision-medicine movement is now shifting back toward personalization, counting on a new ally—the comprehensive, multimodal information that Big Data and AI technologies promise to deliver. On this background, DPM seeks to realize the promise that PM never made. But what exactly is this promise, and can it be taken literally?

Adigozel and Wilson [13] envision a future medicine that will make “truly personalized” health care possible, enabled by mobile and cloud technologies, advanced analytics, and AI. While such statements express aspirations rather than empirical predictions, there are indeed technologies under development that aim to fulfill these hopes. Laino and Savevski [14] identify four major applications that not only strive for individualization but already operate at the level of the individual. These include technologies for (a) prognostic outcomes and risk prediction, (b) clinical diagnostic personalization, (c) therapy development and optimization, and (d) data collection and analysis. The first two domains, in particular, can be regarded as working on personalization rather than mere precision.

- In the area of prognosis and risk prediction, examples include AI imaging and “digital twins” [15]. Earlier PM strategies used risk prediction primarily at the population or subpopulation level, identifying patients who shared specific biological traits. In contrast, new technologies focus on the individual as “one of a kind”. This distinction illustrates what I call *indirect* and *direct personalization*. Personalization is indirect when groups of patients are categorized as most suitable for a given therapy; it is direct when the therapy itself is adjusted to the single patient—even if this remains largely a dream for the future. One might also frame the difference as “personalization through precision” in the former case, and “precision through personalization” in the latter.
- Similarly, clinical diagnostics are becoming personalized in the direct sense, using AI algorithms to analyze a patient’s multimodal data. These data no longer stem solely from clinical sources; they also include “real-world data” (RWD)—information that “comprises attributes that enumerate different important aspects of the way we live our lives” [16]. These data are said to refer to the “real” world for two reasons: first, they are collected outside clinical settings; second, they are intended to reflect patients’ daily lives. RWD can be grouped into four categories: (i) physiological RWD, such as measurements from activity trackers, including step counts and distances walked, or information about a patient’s location obtained through smartphone location services; (ii) psychological RWD, which may capture emotions through the analysis of video recordings or social media posts; (iii) social RWD, which include information on the frequency and duration of phone calls or activities on social media; and, finally, (iv) environmental RWD, which may capture factors such as air quality [16].
- Even though creating a single drug tailored to an individual remains unlikely, up to now, the integration of medical and real-world data now becoming possible could transform pharmaceutical development. One step in this direction is the emerging concept of “cryptopharmaceuticals”. Based on blockchain technology—known mainly from cryptocurrencies but also applied to prevent counterfeit goods—cryptopharmaceuticals go beyond data security. They are pharmaceutical products connected in a patient-specific blockchain of individual dosage units, where each produced dose features a unique, information-rich pattern or cryptographic identifier that is logged to a distributed ledger. They could incorporate a wide range of patient-specific information, such as behavioural patterns and medication history, to inform treatment decisions and dosage adjustments. This approach could move “the pharmaceutical product design principles from mass production of fixed-dose products toward mass customization of information-rich drug products” [17].
- Finally, consider the many wearables used outside the medical context, as well as dedicated medical sensors and transmitters that not only help to engage in “remote control” of patients, but also collect tons of data. These data streams—partly delivered in real time— seem to make genuinely individualized treatment attainable [18].

In sum, medicine first sought greater precision during the early phase of personalized medicine and is now striving for genuine individualization in the age of AI and Big Data. Thus, the objection that PM never claimed to be truly personal applies only to the earlier conception of PM—not to the emerging vision of AI- and data-driven DPM.

## Datafication of patients in the digital era (distortion 1)

If you want to help your patient by using digital technologies, you first need to *extract* data from her and enter it, for instance, into your CDSS—the clinical decision support system, which is now increasingly AI-driven. What could be wrong with that? Physicians have always collected data from their patients. Simply placing your palm on a person’s forehead to feel her temperature is a form of data collection. Data need not be digital. Yet the term *datafication* seems to strike a discordant note with personalised medicine. So, what does it mean, and why might datafication shift the focus away from the individual?

### Some words on datafication

To begin with, *datafication* does not necessarily involve *digitization*, and *digitization* does not equate to *digitalization*. *Datafication* refers to the transformation of aspects of the world into something quantifiable. The result can be analogue. So, you don’t even need a computer to datafy something. *Digitization*, on the other hand, means converting analogue content into digital form, such as when a manually measured height or body temperature is entered into a computer system. Finally, *digitalization* is what we are currently experiencing: “the profound transformation of sectors and societies enabled by digital technologies” [19]. Combining these three concepts and applying them to medicine places us at the heart of today’s challenge. To think that datafication merely means replacing pen and paper with a computer would therefore be a grave misunderstanding of what is happening. The discussion concerns far more than writing a doctor’s report on a word processor.

If datafication does not need to be digital, what, then, is genuinely new about the digital transformation of medicine? One could argue that digitization is simply nothing more than the old-fashioned objectifying datafication of patients—through abstraction, categorization, statistical reasoning, standardization, population-level evidence, or diagnostic reductionism—now just realized in a new medium. This is true to a certain degree. To that extent, digitization and digitalization are only new forms of datafication. However, there is more to it. In particular, there are three aspects that not only add extra speed to the older forms of patient-datafication but also give datafication a largely new quality, turning it into something new.

1.  The first aspect is indeed velocity. The difference between manually collecting, writing down, sorting and analyzing the patient’s clinical parameters, on the one hand, and doing all this digitally within a few seconds is not just an increase in speed. Clinical heuristics are transformed, since the physician’s (or the medical team’s) cognitive tasks are different.
2.  The second aspect regards the phenomena being turned into data: due to sensor technology the amount of collected data increases together with the number of things being datafied. This increase in quantity is accompanied by an increase in the kinds of phenomena regarded as sources. There is hardly any aspect of human life that industry is not trying to convert into data: what you buy in the shopping mall (and when), what your fitness tracker records during your morning run, the messages you exchange with a close friend about your lovesickness, the way you type on your keyboard—all this and much more is seen as a potential source of data. And, of course, your health problems, diagnoses, proposed therapies, prognoses, and—sooner or later—what you verbally communicate to your physician. The entire spectrum of human hopes and fears, plans and actions, weal and woe appear to become quantifiable. Add the velocity of the digital realm, and you have datafication on speed. Everything becomes data that can be processed by computers.
3.  The third aspect that makes digital datafication into something new is its impact on digitalization, i.e. the transformation of large parts of the medical field. As just mentioned, together with the amount and types of phenomena and data the clinical heuristics change, the way physicians do things and solve problems. It is probably not entirely wrong to say that digitization (as digital datafication) is a condition of possibility for digitalization, and that digitalization—in a reverse movement—works as a catalyst for datafication. While the second aspect primarily tends to be a problem of losing the individual patient through "reducing" him to data-packages, this third one may lead to a similar disregard of the individual through a modification of heuristic practices. The first aspect above (velocity) contributes to the other two.

In summary, three points must be taken into account to understand datafication in the digital era: first, that digitization can be regarded as a new evolutionary step in datafication; second, that this (digital) datafication primarily affects the view of the patient; and third, that digitalization primarily affects the physicians (how they do things), and in consequence the patient. Thus, in this regard, we are faced with a tripartite datafication.

### The purported objectivity of data

If we want to clarify why datafication in general can be problematic for individual patient care, it is helpful to remind ourselves of what *data* are—even at the risk of repeating the obvious. It is often assumed that data are *objective*. People say, “It’s true! Just look at the data”. If that were the case, an individual’s unique characteristics could not be lost in the collection of her data. However, this alleged objectivity of data is the first of two central misconceptions that need to be dispelled: data are not objective. Data do not show the world *as it is*. To extract data, one must already have an interest or a question—something one wants to investigate [20]. In this sense, our interest and inquiry already shape and limit what we measure. The same applies to so-called *raw data*. Raw data are just as interest-driven as any other data. Calling data *raw* is an oxymoron, because “data need to be imagined as data to exist and function as such, and the imagination of data entails an interpretative base” [21]. The primary distinction between raw and processed data lies in the point of human intervention: “Raw data” are processed during collection, while “processed data” have been edited after collection [22]. In fact, processed data are processed twice, since data collection itself is a form of processing. Just think of all the physical and mathematical groundwork that must be done before an electroencephalograph can *produce* any data at all. Here lies the root of the big-data “mythology”: the belief that large amounts of data can provide insights and knowledge that were previously unattainable [23]. Data are never fully objective: “If you torture the data enough, nature will always confess” [24]. However, restricting the concept of data to “numbers” would be misleading. Audio and video files may constitute data as well, although their epistemic significance depends on how they are collected, processed, and interpreted.

The second misconception is equating data with *information* or even *knowledge*. While the distinction among the three is well known within the scientific community, it is nevertheless worth recalling that data must be processed and interpreted to become information [21]. From there, information can be used to generate knowledge. Yet even if data are turned into information that leads to knowledge, wisdom does not automatically follow. Russell Ackoff, who added “wisdom” to the DIK (data, information, knowledge) pyramid, explained it thus: knowledge may lead to growth, but wisdom enables development—and development includes an increase in individual value. Since development “is a process in which an individual increases his ability and desire to satisfy his own needs and legitimate desires” [25], wisdom— as a prerequisite for development—is directly relevant to the well-being of the patient, as it entails (at least) balancing short- and long-term interests. Wisdom in this sense is essential for understanding and meeting the needs and desires of persons, which can hardly be understood without taking into account their characteristic qualities. Even though no one can access another individual’s experiences from the inside, neglecting them in the enthusiasm for digitalizing medicine risks an ontological reduction​.

The bedrock of the tripartite datafication sketched above is *dataism*, the belief that everything is quantifiable: according to dataism, what is quantifiable appears objective, while everything else—fervor and fear, love and loneliness—is dismissed as merely subjective, potentially misleading, or illusory [26]. This tends to “swallow” non-quantifiable phenomena. Moreover, we can find a second reverse movement here: while dataism is a major driver for the tripartite datafication, this datafication might, in the eyes of many, vindicate dataism because it is no longer just an idea but a practical reality.

According to Suzuki and colleagues [19], information loss is a consequence inherent particularly to digitization: first during *primary digitization*—the transformation of continuous analogue data into discrete digital data—and then during *secondary digitization*—the representation of this digital data within scoring systems^1^. While this is certainly true, what they do not mention, at least explicitly, is a prior information loss. Before analogue data can be digitized, they must first have been collected. And this collection (or extraction) of data from complex systems such as human beings in their social and environmental living context is already possible only at the expense of real-life information. Medicine is, therefore, faced with at least three points of information loss: (i) datafication, (ii) primary digitization, (iii) secondary digitization. If information loss does, in fact, occur (which I myself tend to assume), the loss will accumulate from (i) to (iii): the scoring systems will process data that are already deprived of certain aspects, and this deprived digital data come from analogue data, which themselves have lost certain aspects of the world.

Against this background, medicine risks losing touch with the individual, suffering patient if it treats data as all it needs to care for patients, thereby overlooking the patient’s lived experience. Once again, this is not merely a matter of losing one category of phenomena and thereby impoverishing medicine. This loss also deprives medicine of the possibility of becoming as personalized as it aspires to be.

### On black boxes and brains

Data and datafication are therefore not inherently objective, disregard the lived experience of the individual, and (to say the least) do not automatically lead to medical wisdom. This is where the opacity of modern AI systems comes into play: the *black-box problem*. The “black box” nature of AI refers to the fundamental difficulty of understanding how AI systems, particularly complex models such as deep neural networks, arrive at their decisions. Although these systems process inputs and produce outputs, the internal mechanisms that connect the two are often opaque, even to their own developers. For example, advanced models can analyze vast datasets and identify patterns beyond human perception. Yet the exact reasoning process, meaning how particular features are selected, weighted, and combined to generate a specific outcome, can be exceedingly difficult, and at times practically impossible, to trace or interpret [27]. This phenomenon is particularly problematic in the medical field, where patients and practitioners alike seek to understand the rationale behind therapeutic decisions. But is this really a problem? Before answering this question, I need to address two commonly made arguments that seem to suggest the answer is no. Let us call the first the *“proof is in the pudding”* argument (see [28] for a concise discussion). This argument posits that the opaqueness of AI systems is not fundamentally different from the (at least partly) unknown causal mechanisms of certain medications.^2^ Sometimes we know that a drug works, but we don’t fully understand how. Since we continue to use drugs that are reliably effective even when their mechanisms are not fully understood, why should we treat reliable but opaque AI systems any differently?

The answer lies in the following consideration: the mechanism of a drug is constant; it does not change. It may have a slightly different effect on you than on me, but that is because you and I are different organisms, not because the drug has “changed its mind”. The situation is markedly different with newer AI techniques. Two kinds of AI systems must be distinguished: *locked* and *adaptive*. In the first case, an AI system is trained, and once it produces satisfactory output, it is locked. The internal algorithm does not change after that, even though we might not know what it exactly does. *Adaptive* or “continuous-learning” systems are different. Based on new incoming data, such a system keeps changing its algorithm even after it has been officially approved. The regulatory challenge that arises from this has been termed the *update problem* [31]. Future CDSS will be of this kind [32]. They are expected to process vast amounts of multimodal data and update their algorithms continuously. Consequently, not only are we unaware of the specific types of data used to generate their outputs, we also do not know their ever-evolving mechanisms of action. The locked system will always produce pudding—let’s hope that the adaptive system does the same. Yet even if the locked system keeps the same pudding-making algorithm, you cannot be sure which ingredients it will use tomorrow: Highly potent CDSSs of the future will be able to automatically search databases of different kinds and from different sources to address a given problem, such as providing a treatment recommendation. Even if the underlying algorithm remains fixed, it is not necessarily the case that new types of data will always be processed in the same way. This significantly distinguishes CDSSs from medications whose mechanisms of action are not well understood but whose effects remain stable. Admittedly, the data sources may also be fixed. Still, the opacity of the system’s algorithms, combined with the vast and potentially varying amount of data processed, makes it difficult to detect errors.

Consider the following two propositions: (a) The system has proved successful for this patient *population*; and (b) The system has proved successful for this *patient*. The distinction between them may appear subtle, yet it is significant. The first proposition can be justified empirically, insofar as its truth is grounded in repeated experience across a plurality of patients. The second proposition cannot be warranted in the same way, since the individual in question has never before undergone treatment according to the AI system’s recommendations. From this perspective, any claim that the individual will in fact benefit amounts not to empirical knowledge but rather to conjecture. The difference may be negligible when the causal mechanism or algorithm is well understood. It may remain negligible even if the algorithm is only approximately comprehended. However, it ceases to be negligible when there is complete ignorance of the algorithm, and it becomes particularly hazardous if the algorithm is dynamic, continuously modifying itself. Moreover, it reflects poorly on medicine if one claims, with London [29], that an advantage of AI networks over the neural networks inside a physician’s skull is that the former can be improved.

I call the second argument against the view that black-box systems are ethically problematic the *“man in the loop*” argument. Bjerring and Busch [33], for instance, do not see medical AI as conflicting with the view of the patient as an individual, since AI is likely to provide only recommendations to the physician, who retains the final decision-making authority and can align the AI’s output with the patient’s individuality. However, the authors overlook that the lack of transparency inherent in AI’s black-box nature—which they themselves acknowledge as a challenge for patient-centered medicine—may hinder precisely this alignment. Such alignment requires understanding both perspectives. While the physician might understand the patient’s viewpoint, she may not fully grasp the rationale behind the AI’s suggestions. This optimism is all the more surprising given that the authors recognize how difficult, if not impossible, it is to understand what AI is doing, in the face of the “millions of weights and thousands of different features” in its neural network and the huge datasets on which it is trained [33]. Apart from potential biases that could unduly influence AI-aided decision-making (a point I address in the next paragraph), it is not unlikely that the physician may be pushed out of the loop by commercial interests—and, partly related to this, by the persistent problem of time pressure in medicine.

To sum up: the first distortion of an individual’s medical characteristics and needs consists of three cumulative factors that might strip the patient of her individuality, reducing her to a faceless member of a patient population. First, data are not objective but always relative to interest-driven processing. Second, data do not yet constitute knowledge and are far from yielding the wisdom a physician requires to care for the uniquely suffering patient. Third, this challenge may be aggravated further still by the possibility that all this remains unnoticed due to the opacity of AI systems.

## Bias (distortion 2)

There seems to be an implicit myth among laypeople that AI systems are virtually infallible, owing to their seemingly “magical powers”. Yet it is wise to remind ourselves that they are not. In the previous section, I discussed those characteristics of datafication that may lead the physician and her decisions away from the individual patient. But the theoretical, technical, and practical challenges—which in turn give rise to ethical problems—do not end with data acquisition. Even if the transformation of a patient’s concerns into discrete digital data were to occur without significant phenomenal or semantic loss, the processing of these data by medical AI and their subsequent use present further challenges that we should at least be aware of.

### On bias

There are many biases.^3^ From research to social relations, from our views of others to even our own self-interpretation—biases are ubiquitous. In principle, we are aware of the possibility of distortions in perception and judgment and are, at least theoretically, able to prevent them. Yet what happens when a database is vast—or even indeterminate—and the algorithm processing it is opaque? This paragraph does not aim to provide a comprehensive overview of all biases relevant to digital medicine, nor to elaborate on strategies to prevent them. Instead, it seeks to emphasize that such biases easily impact the decision-making process concerning the individual patient, thereby shifting the physician’s focus away from him.

But what exactly is a *bias*? Many definitions have been proposed, and it is beyond the scope of this essay to analyze them in detail. For present purposes, it is sufficient to highlight two recurring elements found in the various definitions compiled by Aronson [34]. The first concerns *deviations*, *errors*, or *distortions* from the *truth* or *true outcome*. The second typical element is *systematicity*. Thus, a very (very) brief characterization of bias might be: “A bias is a systematic error.”^4^

In the debate on biases in digital medicine, a third element is often emphasized: the unfair or discriminatory consequences that systematic distortions can have for certain groups (for instance [35, 36]). If an algorithm malfunctions and harms a random group, it may be a tragic error. But if the harm results in an unfair outcome—especially one that systematically affects specific groups—it is likely a bias [37]. Such biases often come at the expense of minorities, particularly certain social or ethnic groups. They are not only epistemic but also moral in nature.

However, an account of bias that focuses exclusively on discrimination seems overly narrow, because biases in medicine can be detrimental even for the general patient population [38]. In this case, no particular group is discriminated against—unless one takes patients altogether as victims. As I will explain in a moment, to focus on the discriminatory features of biases is also inadequate for another reason: the victims of biases in digital medicine may be neither specific groups nor the entire patient population, but rather the patient population seen as a conglomerate of individuals. Since, in the physician’s office, members of groups are not present as collectives but as individuals, it is the individual—not the group—who is ultimately misdiagnosed or mistreated.

To get a handle on the puzzling diversity of biases (and this is all I need for my purposes), the categorization by Friedman and Nissenbaum is helpful [37]. In particular, it shows that not all biases are discriminatory in the narrower sense. The authors differentiate between (a) preexisting bias, (b) technical bias, and (c) emergent bias. Class (a) refers to social and individual cognitive biases that can, in principle, be mitigated through user training. Biases of type (b) are of technical origin and arise, for instance, from hardware limitations or decontextualized algorithms. These biases are still being uncovered, and it remains unclear whether they can be mitigated or whether we must live with them, merely acknowledging their potential influence. One of the technical biases identified by Friedman and Nissenbaum deserves to be cited here, as it goes to the core of our topic: the “Formalization of Human Constructs: Bias that originates from attempts to make human constructs such as discourse, judgments, or intuitions amenable to computers: *when we quantify the qualitative*,* discretize the continuous*,* or formalize the nonformal*” (​[37], italics added). This kind of bias can occur, for instance, when an AI system makes a treatment recommendation based solely on medical calculations, disregarding the ambiguities inherent in human preferences, hopes, and despair. Biases of class (c) arise in the context of use—for instance, when computer algorithms are applied by people for whom they were not originally designed, when new social knowledge emerges that is relevant for the task in question, or when background values have changed.

From a somewhat different perspective, the perhaps best-known typology of biases distinguishes between (1) data-driven bias, when the algorithm may work well but the data are defective; (2) algorithmic bias, when the data may be correct and representative but the algorithm is flawed (due to poor training or human bias); and (3) human bias. Particularly this latter type resembles a whole zoo of outcome-distorting beasts. Among them, we find the automation bias, when the user believes that AI is probably right even if her intuition suggests otherwise. The opposite of this is algorithm aversion bias—the tendency to distrust or reject decisions made by algorithms. Furthermore, there exists confirmation bias, when people tend to trust the machine’s output if it aligns with their own expectations and to refute it if it does not [19, 38]. These are user problems, of course, and cannot be blamed on the machine. All in all, there is a wide range of biases potentially creeping in. Even if we exclude those versions that are purely cognitive in nature or that specifically concern how humans interact with AI systems, there remain enough examples to warrant vigilance. Things get worse when human cognitive biases find their way into an AI’s algorithm—where they are amplified and hidden in the darkness of the machine’s black box.

### Bias and the individual

What does all this mean for the individual? As mentioned above, a distorted output is typically considered a bias when the distortion has a specific character—such as a recurring error pattern. When it results in discriminatory effects, for example against minorities, a particular sex, or economically disadvantaged groups, it is a bias of heightened moral concern. But not all biases are discriminatory, and not every disadvantage experienced by an individual constitutes discrimination in the proper sense. With AI entering medicine, we are now confronted with new types of bias that impact individual patients. These systematically distorted outcomes—such as diagnoses or treatment recommendations—may harm the individual but are not discriminatory. Two such emerging biases are what I call the *Personality Neglect Bias* and the *Homogenization Bias*. They often appear together in the processes of datafication and digitalization, though this is not necessarily always the case. Tendencies to neglect and homogenize characteristic traits of individuals are not new in themselves. However, because they may follow systematically from the tripartite datafication as discussed in this paper, they can be seen as an extension of the existing bias taxonomy.

The *Personality Neglect Bias* refers to the systemic disregard of an individual’s unique psychological makeup, behavioral tendencies, or subjective preferences in data-driven systems. In medical AI, this may manifest when treatment recommendations are generalized without accounting for patient-specific values, coping styles, or communication preferences—potentially reducing adherence or satisfaction with care. This bias results in outcomes that are less effective, unjust, or personally detrimental for that individual. *The Homogenization Bias* arises when AI systems optimize for average outcomes or majority patterns, thereby flattening inter-individual variability. In clinical contexts, this may lead to treatments or diagnostics that work well for the “typical” patient but overlook atypical or minority cases—resulting in suboptimal care. When these two biases co-occur, the individual patient may be doubly disadvantaged: first, by being misaligned with the system’s default assumptions about behavior, values, or needs, and second, by being treated as statistically average. Both these biases seem to be quite similar, and indeed the second one includes the first. Still, they have a structural difference. The Personal Neglect Bias is only this: a systematic neglect of the traits of the individual person that does not need to stem from a homogenizing effect. Although the Homogenization Bias will almost always have a neglect of individual traits as a consequence^5^, the Personal Neglect Bias could instead be due to a general inability of AI systems to reflect and respect individual personality traits. This would result in a non-homogenizing disrespect of, say, individual psychological qualities.

This is also the place to mention “strange errors.” As humans, we all make mistakes. Try multiplying two three-digit numbers in your head, and you’re likely to make an error. Computer systems are better at arithmetic than you or me. Yet they, too, can make errors. The problem with AI systems, however, is that they sometimes make such bizarre mistakes that a human would lose his job for committing the same error. The internet, for instance, is full of weird answers people have received from LLMs (large language models). Similar egregious errors are known from image-classifying AI, such as mistaking a squirrel for a sea lion or a dragonfly for a manhole cover. What makes errors like these particularly troubling is not their strangeness alone, but the potentially serious harm they may inflict on the patient [39].

## Distancing and virtualization (distortion 3)

An important element of medicine’s ongoing digitalization project is *telemedicine*. There are several related terms in use, including *telehealth*, *telecare*, and *mHealth*, each with a specific focus on applications and technologies, and each presenting distinct ethical challenges. However, the exact meanings and differences between these terms are not the primary concern here; rather, it is the shared aspect of *distancing* that is of interest. Their tendency to distance and virtualize risks making the patient “unreal”, in a sense. Therefore, I follow Lupton’s definition and use “telemedicine” as an umbrella term that “involves the use of digital technologies by healthcare providers to communicate with patients and other providers, effect clinical diagnoses and deliver healthcare in remote locations. It also includes patient self-care and self-monitoring systems using digital technologies” [40].^6^ Telemedicine is not restricted to video-based encounters between physicians and patients, as was particularly common during the COVID-19 pandemic. In principle, the concept encompasses all health-related practices, both direct and indirect,^7^ that occur over a spatiotemporal distance. Thus, the range of possible applications of telemedicine is wide. It ranges from telephone consultations with a family doctor to the use of “smart pills” (for example, pills that can “notify” a physician through an electronic transmitter when they have been taken.); from web-based patient evaluations and pretransfer screenings to virtual consultations for primary care; and from home support services to remote diagnostic expert assistance [41]. All this can assist healthcare workers, make medical workflows more efficient, and reduce costs. Not least, telemedicine can be to the benefit of patients.

Yet, history has shown time and again that things we initially welcome with enthusiasm often turn out to be far from unproblematic. Regardless of its blessings, telemedicine also carries the risk of reducing the flesh-and-blood patient to an abstract and disembodied heap of data. As Ferrarello puts it: “Each problem becomes a cognitive one without a context or body” [42]. Since much has been said on the ethics of telemedicine in general, I will examine three exemplary “losses” that may result from the distancing and virtualization of patients, physicians, and care itself. These are the *loss of the (individual) body*, the *loss of integrated locus and focus*, and the *loss of context*. These losses intersect and reinforce one another. Nevertheless, each one has a specific focal point. Together, and in tandem with datafication and bias, they could render the patient invisible as an individual to medicine.

### Loss of the (individual) body

Telemedicine is not merely medicine at a distance, or medicine without the physical presence of a body. If that were the case, one might suspect it to be a kind of reduced or “shrunken” version of medicine, where the shrinking consists in a mere quantitative decrease of information caused by the absence of the body. In reality, thanks to technologies that enable multifaceted, continuous, 24/7 remote monitoring, medicine is now arguably more comprehensive than ever in quantitative terms. However, this increased quantity and precision relates primarily to the *physical body* rather than the *lived body*. The phenomenological distinction between the objective, measurable body as produced and represented by medical technologies through numbers and images, and the lived body experienced by the patient as a subject, is of vital importance for a humane medicine. This does not mean that technology necessarily reduces the patient to a biological something rather than recognizing him as an individual. Svenaeus [43], for example, distinguishes between “good and bad forms of objectification in medicine.” Yet, since the lived body is primarily defined by the individual *experiencing* her physical body, what telemedicine risks missing is a whole category of information—the particular qualities of a particular being: in other words, aspects of that being’s individuality. Whether telemedical contact occurs via text messages, video consultations, remote transmission of sensor data from the patient’s home, or even through a digital twin, each case lacks essential information. The degree of information loss varies, of course, with the respective technology: a chart of the patient’s vital signs is more abstract than a video call between a patient and a physician who have known each other for many years. In this regard, it would be unfair, and plainly wrong, to charge telemedicine as a whole with losing contact to the individual patient. To avoid this, it would be worthwhile (yet beyond the scope of this article) to sort diverse practices—such as video and telephone consultations, asynchronous messaging, remote monitoring through sensor-based medicine, or digitally mediated chronic disease management—along a scale. This scale would reach from a video call between a patient and physician who are already familiar with each other on one end, to remote monitoring of the patient’s clinical parameters by someone who has never seen or spoken with the patient in person on the other end. Moreover, combining the different technologies in varying assemblies and incorporating their respective impact on patient understanding will lead to a multidimensional chart that can help avoid the losses in question.

What certain forms of telemedicine may risk losing can be illustrated through Lupton and Maslen, who stress that medical work is “body work, involving patients’ and practitioners’ mutual constitution of bodies as medical objects and tools via specific embodied practices” [44]. This involves touching the patient’s body, listening to bodily sounds and the tone of the patient’s voice, and even using smell to detect signs of infection. The absence of such practices and phenomena can, but does not necessarily, result not only in missed diagnoses [45] but also in the loss of subtle cues that reveal how *this particular patient* is coping with her illness.

Another ethically significant aspect of the presence or absence of the individual body concerns communication. Physicians and patients usually meet as strangers. As most of us learned during the COVID-19 pandemic, when communication was often limited to video meetings, it is very difficult—if not impossible—to become acquainted with someone *solely* through small video tiles or text messages. The same holds true in medical contexts. Without the directly present (lived) body and the nonverbal cues so crucial to the physician–patient relationship, communication tends to be deficient.^8^ “Nothing in medicine is more compelling than the living human face of a patient in need” [47].

### Loss of integrated locus and focus

Of course, even in telemedicine, the patient’s body does not cease to exist. Yet, as the prefix *tele* indicates, it is no longer where it used to be—namely, with the caring physician. The patient’s remoteness results in three principal forms of fragmentation: (a) the locus of care is divided; (b) medical activities are redistributed; and, as a consequence, (c) the focus on the suffering individual becomes blurred. These fragmentations are interconnected; together they constitute a single phenomenon viewed from different perspectives.

-  Traditionally, the locus of care was the patient being physically present with their physician—in the physician’s office, the hospital room, or at the patient’s home. All involved agents, data, measures, and devices were in one place. In addition, the necessary decisions were made within this framework [48]. Admittedly, the disaggregation of this setting began decades ago with the advent of modern information technologies. With telemedicine, however, the frequency and degree of disintegrated treatment situations have increased rapidly. In a remote monitoring system, for instance, data collection, analysis, and medical advice become uncoupled [49].
- This breakdown both results from and necessitates a redistribution of work [44]: patients (i) take their own measurements, (ii) do so alone or (iii) with assistance from a technical expert or nurse, (iv) communicate remotely with the physician, who (v) may then seek advice from an AI system.
-  Apart from challenges regarding the distribution of responsibility, medicine should recognize that the focus on a patient’s individual characteristics and needs may be neglected in such a situation. The splitting of what I call the *spatiotemporally integrated treatment situation* is not by itself disastrous, of course. But it may have negative consequences if helping a patient means helping *this particular* patient, and not merely some member of a patient population.

### Loss of context

The term “context” can refer to different things: it may describe the patient’s personal circumstances, or the circumstances under which her medical data have been obtained. In a third sense, it may also refer to those medical data that have been omitted. No data representation of a patient is exhaustive; much is necessarily left out, and the physician’s view of a remote patient is therefore necessarily restricted and incomplete. This can easily lead to a decontextualization of the patient’s condition, where she loses control over how her health problems are understood by clinicians and carers [50]. Losing such contextual information may, as a consequence, lead to what Knobel termed “ontic occlusion.” According to Knobel, ontic occlusion takes place when a certain representation of a phenomenon, idea, or situation takes precedence and blocks (occludes) another representation. The blocked elements can thus no longer enter into the discourse, while the exclusion itself disappears from the explanatory scene. As a result, we are left with something as “objective” that is not [51]. Since not only the blocked elements disappear but the blocking itself, we become blind to errors. Although problems such as ontic occlusion primarily arise in the context of big data (see [52], or [53] for instance), they are becoming increasingly relevant for patient care. They do not occur when interviewing a patient face-to-face, but must be considered when incorporating “real-life data”—for example, from sensors or patients’ online posts—into the anamnesis, while the patient is physically or mentally not present.^9^ This applies *a fortiori* when the AI analysis of a CDSS encompasses the records of thousands of other patients. Bush discusses no fewer than twelve ways of losing something in the building of large-scale data sets. As mentioned, telemedicine is not the same as big data, but the mechanisms of losing contextual information are similar. As Bush puts it: “[A]ll technoscientific work […] requires simplification. Problems arise when, even as we simplify so as to make problems tractable, we forget such simplification and treat the results of our analyses as brute facts” [54]. In summary, telemedicine not only risks losing important contextual information about the patient but also tends to overlook that loss.

Telemedical losses such as these have profound implications for patient care. If the patient disappears as an individual, becoming merely a representative of a generalized population, whose members are passably similar, individualized care and counselling become harder. In addition, deskilling can result from lack of practice, as AI assumes more tasks, or from fewer occurrences of phenomena owing to the deindividualizing mechanisms discussed in this essay.

## Conclusion

Few would seriously doubt that new digital technologies are ushering medicine into a new era of research, care, and cure—whether through the analysis of multimodal datasets enabled by big data or through insights generated by artificial intelligence. The hope is to move beyond the “one-size-fits-all” model toward a form of medicine that tailors treatment to each patient’s individuality. Yet this essay invites physicians and ethicists alike to pause and reflect, for developments might not unfold as anticipated. The very technologies expected to advance individualized care could, paradoxically, lead medicine further away from the individual—even though this is not inevitable.

I have identified three areas of concern—or “distortions”, as I call them—each encompassing several specific subproblems. The first distortion can arise from datafication. Contrary to a common assumption, data are not “objective”; they are always already mediated by processes of selection and interpretation, potentially omitting precisely what makes each patient unique. Moreover, translating continuous analogue phenomena into discrete digital representations almost inevitably entails a loss of meaning. Even if data were not preformed, they remain far removed from knowledge or wisdom—qualities indispensable for recognizing and responding to the person before the clinician. These limitations are easily obscured by the opaque, black-box character of medical AI systems.

The second distortion lies in the area of bias. Although bias in digital medicine is widely acknowledged (as in science generally), its ethical significance for the individual patient remains underexplored. Bias is typically discussed in relation to group-level discrimination, yet its effects are also deeply personal. The many forms of bias converge in what might be called “Personality Neglect Bias” and “Homogenization Bias,” both of which threaten to erode the moral and clinical attention owed to each unique patient.

The third distortion concerns telemedicine, whose inherent tendency to distance and virtualize the patient risks attenuating the relational and embodied dimensions of care that are central to medical ethics. Video-based meetings as such may preserve many of the phenomenal qualities of traditional face-to-face encounters in suitable situations; yet together with more abstract elements of telemedicine, the virtualization tendencies can become detrimental for both the physician and the patient.

What does this imply for the emerging AI- and data-driven medicine that is reshaping the moral character of the field? My aim was not to diminish the promise of technological innovation or to claim that such systems cannot serve human health. Rather, it was to caution that the very tools designed to fulfil the implicit promise of personalized medicine might, in practice, risk achieving the opposite: the loss of the individual. If medicine is not aware of this risk, it is in danger of losing sight of what could be circumscribed as the perennial nucleus of the medical endeavour: the care for an individual, suffering being. Care is not limited to cure and the relief of pain. Care includes—if not presupposes—empathy, the embodied encounter, narrative understanding, individualized judgment, moral recognition, and clinical wisdom (which is more than just technical judgment). This is what turns medicine from a service into something deeply humane.

## Data Availability

No datasets were generated or analysed during the current study.

## Footnotes

1 It should be noted that scoring systems have been common in medicine for a long time and do not need to be digital. The information loss characteristic of them is therefore not (primarily) due to their being digital.

2 The argumentation comes in variations. Another one could be named the ‘black brain argument’, claiming that the brain of the physician is no less opaque than AI [29, 30].

3 See for example the “Catalogue of Bias”, where currently 65 examples are listed: https://catalogofbias.org/.

4 I do know that I’m on rather thin ice with this proposal.

5 The homogenization would not neglect individuality if the person in question happens to have exactly average traits. But this is only a theoretical possibility.

6 The only difference to Lupton’s use of the term is that she includes online medical education and I don’t.

7 Fitness trackers, for instance, are indirectly health-related. While they are primarily intended for personal use, they can also be used for medical purposes.

8 Grīnfelde [46] suggests the opposite. According to her, it is the physician’s “internalized clinical gaze” during traditional in-person visits that objectifies the patient as something that needs to be fixed. Only the telemedical encounter, with the body being remote enough, compels the physician to view the patient as a person and to listen to her narrative. I do not think this can be generalized. There may well be physicians who objectify their patients through the clinical gaze. But it is—at least to my mind—not convincing to assume that medicine as such is inherently of that nature, and—even if that were the case—that telemedicine would be the remedy.

9 It might seem as if ontic occlusion cannot occur with real-life data, since such data are, by definition, ‘real.’ Yet this is a misconception. ‘Real’ only indicates that the data are collected outside the clinical setting, i.e., in ‘real life’; it does not mean that they are any closer to reality than other data.
